# Rituximab combined with lenalidomide for the treatment of marginal zone lymphoma with IgM kappa positivity and cold agglutinin syndrome: a case report

**DOI:** 10.3389/fimmu.2026.1780180

**Published:** 2026-04-07

**Authors:** Xueya Zhang, Yuyu Zheng, Suqin Cai

**Affiliations:** 1 Department of Hematology, The Second Affiliated Hospital of Fujian Medical University, Quanzhou, Fujian, China; 2 Rare Disease Medical Center, The Second Affiliated Hospital of Fujian Medical University, Quanzhou, Fujian, China; 3 Department of Clinical Laboratory, The Second Affiliated Hospital of Fujian Medical University, Quanzhou, Fujian, China; 4 Department of Pathology, The Second Affiliated Hospital of Fujian Medical University, Quanzhou, Fujian, China

**Keywords:** cold agglutinin syndrome, IgM kappa, lenalidomide, marginal zone lymphoma, rituximab

## Abstract

Cold agglutinin disease (CAD) and cold agglutinin syndrome (CAS) are currently recognized as distinct clinical entities. CAD is defined as a distinct clonal B-cell lymphoproliferative disorder of the bone marrow. CAD is also a subgroup of autoimmune hemolytic anemia, triggered by cold-reactive immunoglobulin targeting red blood cell surface antigens in its pathogenesis. CAS exhibits similar cold hemolytic anemia; however, it is secondary to hematological malignancies or infectious diseases. For lymphoma-associated CAS, specific types of antilymphoma chemotherapy are required. We report a man in his late 60s with marginal zone lymphoma with IgM kappa positivity and CAS. The patient had no response following two cycles of rituximab monotherapy administered at 375 mg/m2 (2) on day 1 every 21 days. He was then treated with a regimen consisting of rituximab 375 mg/m2 (2) on day 1 and lenalidomide 20 mg daily on days 1–21, administered every 28 days. After four cycles of treatment, the patient’s cold agglutinin test turned negative, and no red blood cell agglutination was observed in peripheral blood and bone marrow. After completing six cycles of rituximab and lenalidomide therapy, the condition has remained in complete remission for 24 months. CAS is rare in clinical practice, and effective treatment for underlying diseases is key to improving patient prognosis. The combination therapy of rituximab and lenalidomide is one worthy of clinical practice as a salvage regimen for such diseases, especially for those who are refractory to rituximab monotherapy.

## Background

Cold agglutinin disease (CAD) and cold agglutinin syndrome (CAS) are currently recognized as distinct clinical entities. CAD is defined as a distinct clonal B-cell lymphoproliferative disorder of the bone marrow. CAD is not simply a subgroup of autoimmune hemolytic anemia, triggered by cold-reactive immunoglobulin targeting red blood cell surface antigens in its pathogenesis; rather, it represents cold antibody-mediated autoimmune hemolytic anemia (cold AIHA) (1). Primary CAD is a clonal B-cell disease characterized by clonal expansion of B cells in the bone marrow (2). In contrast, CAS exhibits similar cold hemolytic anemia; however, it is secondary to hematological malignancies. For example, non-Hodgkin’s lymphoma, including marginal zone lymphoma, is present in approximately 9% of CAD cases (3). In infectious diseases, it is especially common in *Mycoplasma pneumoniae* and EB virus infections (4). Lymphoma-associated cold agglutinins are monoclonal and often do not resolve spontaneously; moreover, they do not respond to glucocorticoids or splenectomy. Consequently, a regimen based on rituximab is employed to eradicate the pathological B-cell clones that produce monoclonal cold agglutinins in the bone marrow. However, trial reports on the use of rituximab at a dose of 375 mg/m2 (2) per week for 4 weeks show a response rate of 45% to 58%, with only a minority of patients achieving complete response (5). The likelihood of sustained response is low, with 57% to 89% of responding patients eventually relapsing (5). Thus, specific types of antilymphoma chemotherapy are required. Herein, we describe a case of marginal zone lymphoma with IgM kappa positivity and CAS who had no response to rituximab monotherapy. Afterxreceiving a combination therapy of rituximab 375 mg/m2 (2) on day 1 and lenalidomide 20 mg on days 1–21 therapy for four cycles, administered every 28 days, the patient achieved complete remission.

## Case

**Figure 1:**
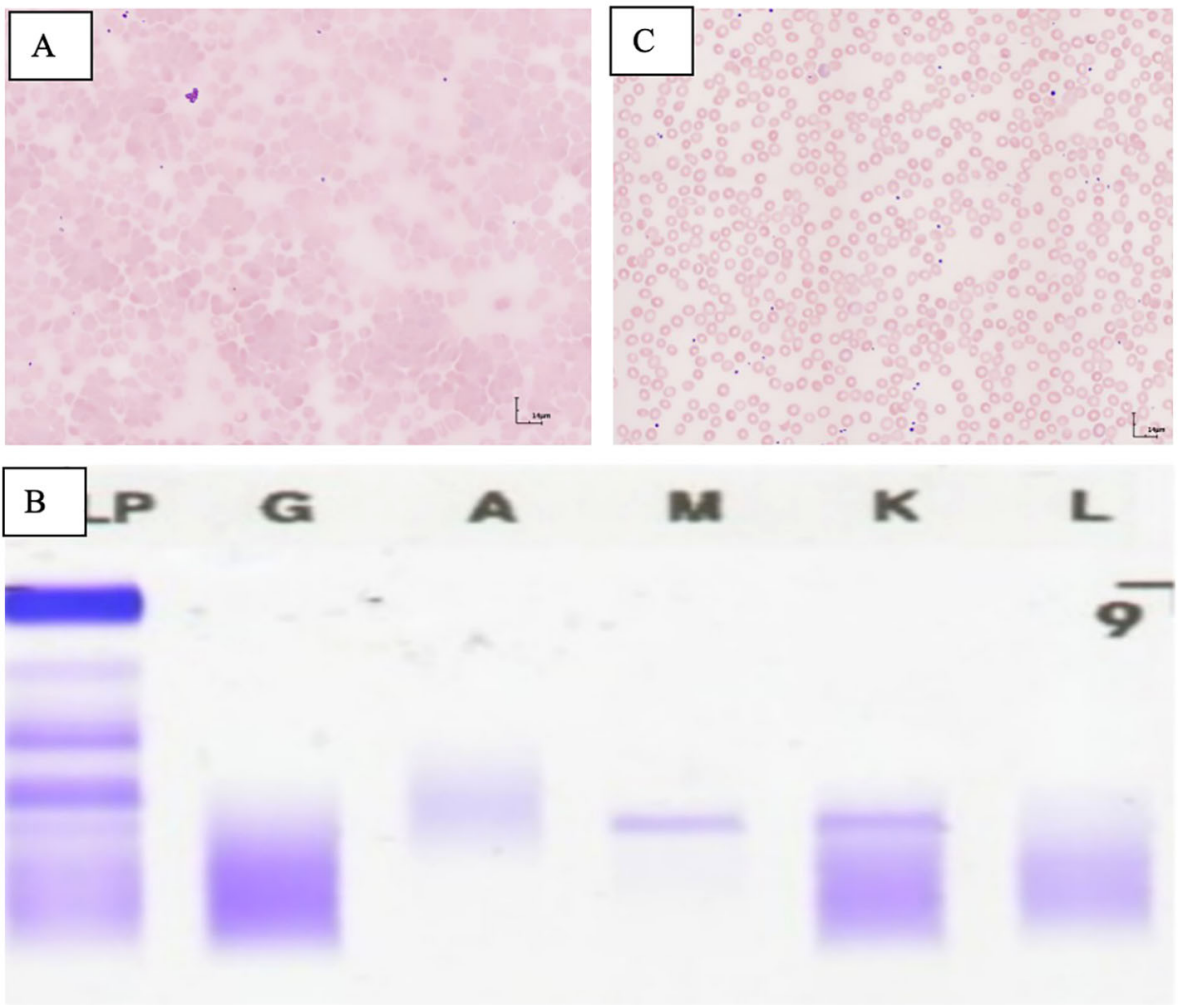
**(A)** Visible red blood cell agglutination phenomenon under a microscope (×400). **(B)** Immunofixation electrophoresis analysis suggested that immunoglobulin M and kappa (IgMκ) were positive. **(C)** No red blood cell agglutination was observed in peripheral blood (×400).

**Figure 2:**
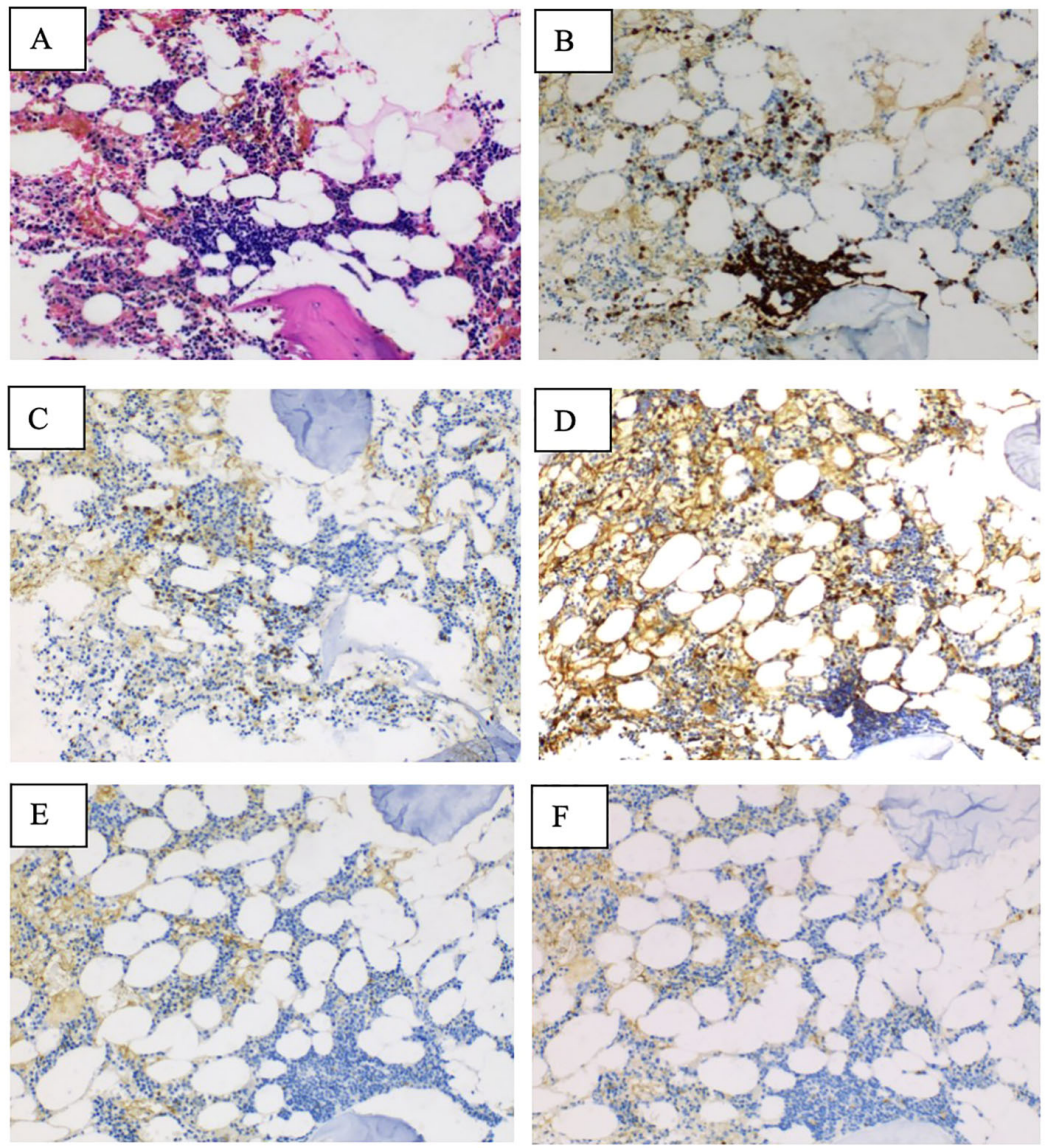
**(A)** Immunohistochemical staining results of bone marrow marginal zone lymphoma (hematoxylin–eosin staining ×100). (**B–F**) The results of CD20, CD5, CD10, CD23, and Cyclin D1 expression (EnVision method ×100).

A man in his late 60s was admitted in mid-2023, with the main complaint of peripheral cyanosis of the limbs for more than 2 months. No relevant comorbidities or any prior treatments have been recorded in history. Physical examination did not reveal abnormalities. The patient’s performance status was moderate. Blood routine examination indicated the following results: white blood cell count 4.9 × 10^9^/L (normal range: 3.5–9.5), neutrophils 2.03 × 10^9^/L (normal range: 1.8–6.3), hemoglobin 137 g/L (normal range: 130–175), and platelets 201 × 10^9^/L (normal range: 125–350), with visible red blood cell agglutination under the microscope (**Figure 1A**). Biochemical results showed albumin 44.2 g/L (normal range: 40–55), total bilirubin 8.88 μmol/L (normal range: 0–26), indirect bilirubin 3.4 μmol/L (normal range: 0–20), lactate dehydrogenase 159.3 IU/L (normal range: 120–250), alanine aminotransferase 11.6 U/L (normal range: 9–50), aspartate aminotransferase 14.8 U/L (normal range: 15–40), creatinine 93.4 μmol/L (normal range: 57–97), and uric acid 439 μmol/L (normal range: 202–416). Haptoglobin was 0.8 g/L (normal range: 0.5–1.6). The cold agglutination test was positive with a titer of 1:512. Immunofixation electrophoresis analysis suggested that immunoglobulin M and kappa (IgMκ) were positive (**Figure 1B**). Free kappa light chain was 96.35 mg/L, free lambda light chain was 20.64 mg/L, and kappa/lambda was 4.668. The bone marrow smear showed obvious agglutination of mature red blood cells. Bone marrow biopsy demonstrated that CD20, CD42b, CD235a and MPO were positive, while CD3 CKpan, CD23, CD5, Cyclin D1, CD10, Bcl-2, Bcl-6, CD138, Kappa, and Lambda were negative (**Figures 2A–F**). Moreover, BRAF V600E, MYD88 L265P, and CXCR4 gene mutations were negative. However, IgVH, IgDH, and IgK gene rearrangements were detected. According to diagnostic and treatment standards (6), the patient was diagnosed with extranodal marginal zone lymphoma with IgM kappa positivity and cold agglutinin syndrome. The patient then received rituximab monotherapy at a dose of 375 mg/m2 (2) on day 1 for two cycles, administered every 21 days. However, the cold agglutinin titer did not decrease, and red blood cell agglutination was observed in peripheral blood, suggesting no response to rituximab monotherapy. Thus, he was treated with a regimen consisting of rituximab 375 mg/m2 (2) on day 1 and lenalidomide 20 mg daily on days 1–21, administered every 28 days. No adverse events were observed during the rituximab–lenalidomide combination therapy. After four cycles of treatment, the patient’s cold agglutinin test turned negative (**Table 1**), and no red blood cell agglutination was observed in peripheral blood (**Figure 1C**). The patient’s symptom of peripheral cyanosis of the limbs improved, and his performance status was better. Re-examination of the bone marrow showed no red blood cell agglutination, and bone marrow biopsy suggested normal bone marrow proliferation. After completing six cycles of treatment with rituximab combined with lenalidomide, the patient entered the follow-up observation period. To date, the patient’s condition has remained in complete remission for 24 months.

**Table 1:** The medication timing and regimen during the patient’s treatment process and the results of the cold agglutinin test.

Medication time	Cold agglutinin test	Therapeutic regimen
Before 1 cycle	1:512	Rituximab
Before 2 cycles	1:512	Rituximab
Before 1 cycle	1:512	Rituximab + lenalidomide
Before 2 cycles	1:256	Rituximab + lenalidomide
Before 3 cycles	1:128	Rituximab + lenalidomide
Before 4 cycles	Negative	Rituximab + lenalidomide
Before 5 cycles	Negative	Rituximab + lenalidomide
Before 6 cycles	Negative	Rituximab + lenalidomide
3 months after 6 cycles	Negative	Follow-up observation
6 months after 6 cycles	Negative	Follow-up observation
9 months after 6 cycles	Negative	Follow-up observation
12 months after 6 cycles	Negative	Follow-up observation
18 months after 6 cycles	Negative	Follow-up observation
24 months after 6 cycles	Negative	Follow-up observation

## Discussion

CAD is an important type of autoimmune hemolytic anemia with cold antibodies, including primary CAD and secondary CAS (7). In addition to symptoms of hemolytic anemia, more than 90% of patients with CAD may present with peripheral circulatory symptoms such as cold-induced acrocyanosis, livedo reticularis, and Raynaud’s phenomenon (3). The main manifestations of the patient are peripheral cyanosis of the limbs, which subsides after warming, and mild limitation of movement during cold exposure, but without anemia symptoms, lymphadenopathy, or hepatosplenomegaly.

Based on the negative signs and classic symptoms, we focused on observing the morphological characteristics of red blood cells, which suggested that significant red blood cell agglutination can be seen in both peripheral blood and bone marrow samples. Moreover, red blood cell autoantibodies indicated a positive cold agglutinin test with a titer of 1:512.

Cold agglutinins are present in both CAD and CAS; however, the cells that produce cold agglutinins are monoclonal in CAD and CAS secondary to lymphoma, whereas they are polyclonal in CAS secondary to infection (8). Immunofixation electrophoresis analysis suggested that monoclonal immunoglobulin M and kappa (IgMκ) were positive in the current patient’s blood sample. The monoclonal IgMκ is specific to red blood cell surface antigens, leading to a series of cold agglutinin-related symptoms of chronic hemolytic anemia and signs of cold-induced acrocyanosis. Moreover, the clinical manifestations of CAS are related to the underlying disease (4).

Previous studies reported that CAD and CAS could be related to low-grade lymphoma (9) and splenic marginal zone B-cell lymphoma (10). Therefore, to investigate for potential diseases, bone marrow samples were obtained from the patient with his informed consent. Abnormal B lymphocytes were detected by flow cytometry immunophenotyping, with positive expression of HLA DR, CD19, CD20, CD22, CD200, sKappa, and Bcl-2, without enlargement of the lymph nodes, liver, and spleen, suggesting a diagnosis of extranodal marginal zone lymphoma confirmed by bone marrow biopsy. However, BRAF V600E, MYD88 L265P, and CXCR4 gene mutations were all negative, which was not consistent with the diagnosis of Waldenström macroglobulinemia and lymphoplasmacytic lymphoma. Moreover, Ig gene rearrangement fragment analysis results indicated that IgVH, IgDH, and IgK gene rearrangements were positive. Thus, the patient was ultimately diagnosed with extranodal marginal zone lymphoma with IgM kappa and CAS.

The treatment of CAD includes non-pharmacological and pharmacological treatment. When non-pharmacological treatment is ineffective or there are serious circulatory system symptoms that affect daily life, pharmacological treatment can be chosen (11). Drug therapy includes treatment targeting B cells and treatment with complement inhibitors. The treatment for B cells that produce CA is mainly based on rituximab, with a complete remission rate of less than 5% for monotherapy (3). Combined with bendamustine or fludarabine, the complete remission rate can be increased to 20%–40%, but there are risks of bone marrow suppression, granulocyte deficiency, and infectious fever. We administered rituximab monotherapy for two cycles; however, the titer of cold agglutinin did not decrease, and red blood cell agglutination was also observed in peripheral blood, suggesting that the patient had no response to rituximab monotherapy. Lenalidomide is an immunomodulatory agent, with direct antitumor activity and immune synapse modulation, including activation of T and NK cells (12). Moreover, this could be relevant for targeting the clonal B-cell process in marginal zone lymphoma-associated CAS, considering the efficacy and tolerability of the rituximab–lenalidomide combination in various lymphomas, especially regarding tolerability in an older patient (13). Thus, the patient received rituximab combined with lenalidomide chemotherapy. After four cycles of treatment, the patient’s cold agglutinin test turned negative, and no red blood cell agglutination was observed in peripheral blood. Re-examination of bone marrow showed no red blood cell agglutination, and bone marrow pathology suggested normal bone marrow proliferation without lymphocyte increase. At the same time, there were no adverse reactions such as granulocyte deficiency, infection, or fever. At present, the patient has been followed up for 24 months after completing the six cycles of rituximab combined with lenalidomide, and the condition is still in complete remission.

We describe a case of marginal zone lymphoma with IgM kappa positivity and CAS who had no response to rituximab monotherapy, but achieved long-term complete remission after treatment with rituximab combined with lenalidomide. Cold agglutinin syndrome is rare in clinical practice, and effective treatment for underlying diseases is key to improving patient prognosis. The combination therapy of rituximab and lenalidomide is one worthy of clinical practice for salvage regimen of such diseases, especially for those who are refractory to rituximab monotherapy.

## Data Availability

The raw data supporting the conclusions of this article will be made available by the authors, without undue reservation.
